# Dorsal Anterior Cingulate Thickness Is Related to Alexithymia in Childhood Trauma-Related PTSD

**DOI:** 10.1371/journal.pone.0139807

**Published:** 2015-10-06

**Authors:** Lauren A. Demers, Elizabeth A. Olson, David J. Crowley, Scott L. Rauch, Isabelle M. Rosso

**Affiliations:** 1 Center for Depression, Anxiety and Stress Research, McLean Hospital, Belmont, Massachusetts, United States of America; 2 Department of Psychiatry, Harvard Medical School, Boston, Massachusetts, United States of America; Univ of Toledo, UNITED STATES

## Abstract

Alexithymia, or “no words for feelings”, is highly prevalent in samples with childhood maltreatment and posttraumatic stress disorder (PTSD). The dorsal anterior cingulate cortex (dACC) has been identified as a key region involved in alexithymia, early life trauma, and PTSD. Functional alterations in the dACC also have been associated with alexithymia in PTSD. This study examined whether dACC morphology is a neural correlate of alexithymia in child maltreatment-related PTSD. Sixteen adults with PTSD and a history of childhood sexual abuse, physical abuse, or exposure to domestic violence, and 24 healthy controls (HC) completed the Toronto Alexithymia Scale 20 (TAS–20) and underwent magnetic resonance imaging. Cortical thickness of the dACC was measured using FreeSurfer, and values were correlated with TAS–20 scores, controlling for sex and age, in both groups. Average TAS–20 score was significantly higher in the PTSD than the HC group. TAS–20 scores were significantly positively associated with dACC thickness only in the PTSD group. This association was strongest in the left hemisphere and for TAS–20 subscales that assess difficulty identifying and describing feelings. We found that increasing dACC gray matter thickness is a neural correlate of greater alexithymia in the context of PTSD with childhood maltreatment. While findings are correlational, they motivate further inquiry into the relationships between childhood adversity, emotional awareness and expression, and dACC morphologic development in trauma-related psychopathology.

## Introduction

Alexithymia refers to deficits in identifying, labeling, and communicating one’s emotional state [1,2,3]. One correlate of elevated alexithymia is exposure to traumatic life events, particularly childhood trauma [4]. Krystal [5] provided one of the first descriptions of this psychological profile in trauma-exposed individuals, and proposed that posttraumatic alexithymia arises from an uncoupling of cognitive and affective processing in response to the overwhelming emotional intensity of a traumatic experience. In fact, the prevalence rate of alexithymia among individuals with posttraumatic stress disorder (PTSD) has been cited to be as high as 85% [6]. Subsequent research has confirmed that alexithymia is highly prevalent in PTSD, and that alexithymic PTSD patients experience intense emotions that are poorly controlled by higher-order cognitive processes [7–9].

For individuals with a history of childhood maltreatment, alterations in typical neurodevelopmental processes likely mediate the relationship between trauma and alexithymia. In a study by Zlotnick and colleagues [9], alexithymia was more prominent in outpatients with PTSD than outpatients with other psychiatric disorders, and physical and emotional neglect during childhood were the only types of trauma independently associated with severity of alexithymia. There is also evidence that alexithymia commonly occurs in individuals with major depressive disorder (MDD) [10], and that childhood emotional abuse and neglect predict later manifestation of alexithymia and somatic symptoms in depression [11]. Studies of nonclinical populations also have found that childhood abuse predicts deficits in emotional awareness and expression (alexithymia) [12]. Taken together, these findings are consistent with hypotheses that maltreatment during childhood alters or impedes the development of emotional processing skills, the ability to integrate thoughts and feelings, and/or the capacity to express and regulate emotions [4,5]. Neuroscience research has shown that early life stress may adversely affect brain development through mechanisms such as alterations in developmentally appropriate synaptic elaboration and pruning [13,14]. Moreover, an effect of early trauma on synaptic connectivity patterns has been demonstrated in brain areas involved in emotional appraisal and regulation, including the anterior cingulate cortex (ACC; [14])

The ACC, particularly its dorsal or posterior extent, has been implicated as a key cortical correlate of alexithymia [15]. The dorsal division of the ACC (or anterior mid-cingulate) is involved in a breadth of psychological functions, and it is reliably engaged by emotional stimuli as well as tasks requiring cognitive control [16]. Moreover, variation in both the morphology and function of the dorsal ACC (dACC) has been associated with individual differences in alexithymia, consistent with the definition of alexithymia as a deficit in the cognitive evaluation and control of emotions. In anatomical studies using magnetic resonance imaging (MRI), increased gray matter volume of the dACC has been associated with greater attention to emotions and regulation of negative feeling states [17] and with greater cognitive reappraisal of emotion [18]. Findings from MRI studies of alexithymic participants have reported larger dACC [19], smaller dACC [20], or no dACC volume difference compared with low alexithymic participants [21]. In a recent morphometric study, decreased gray matter volume of the dACC was associated with elevated alexithymia in a general population sample (*N =* 1,685) [22]. Likewise, in functional imaging studies, alexithymics have shown increased [23,24] but also decreased activation of the dACC [25–28] when processing emotional stimuli. Differences in the directionality of findings may relate to the valence and arousal level of stimuli [27,29]. As a whole, this body of research suggests that the dACC is related to alexithymia, consistent with this brain region’s role in conscious emotional processing and integration of cognitive and emotional stimuli [16,30,31].

A seminal meta-analysis of functional neuroimaging studies identified the dorsal ACC as central to PTSD pathophysiology [32]. Moreover, the dACC is also particularly sensitive to the effects of trauma during childhood. Reductions in gray matter density or volume of the dACC have been found in patients with child abuse-related PTSD compared with nontraumatized controls [33] and in adults with adverse childhood experiences broadly defined [34]. Neuronal integrity, as measured by the ratio of N-acetylaspartate to creatine, in the dACC is reduced in maltreated children compared to non-maltreated children [35]. Additionally, in a study of structural brain connectivity, the left ACC had a significantly less complex connectivity pattern and fewer anatomical connections with other brain regions in young adults with histories of maltreatment than in unexposed control subjects [36]. Altogether, there is strong evidence of the dACC’s involvement in mediating some of the effects of childhood trauma and related posttraumatic psychopathology.

Thus far, research evaluating the neural correlates of alexithymia in PTSD has used functional imaging approaches. In two studies using trauma-related script imagery, PTSD patients with high alexithymia scores showed reduced activity in the ACC compared with PTSD patients with low alexithymia scores [37,38] and this pattern also was evident in trauma-exposed adults with and without alexithymia [37]. Alexithymia predicted significantly greater activation during this symptom provocation task in the bilateral insula, posterior cingulate cortex, and thalamus–all brain regions implicated in emotional awareness and regulation [37]. Interestingly, in another study from the same research group, individual differences on a measure of emotional awareness were differentially correlated with ACC function in PTSD patients and trauma-exposed healthy subjects during trauma-related script imagery [39]. Specifically, emotional awareness correlated positively with a large cluster of ACC activity in healthy adults. In contrast, emotional awareness correlated negatively with activity that localized to a more ventral ACC cluster among PTSD patients. While these findings implicate dACC function in alexithymia in PTSD patients, at this time it is unclear whether there are associated morphological alterations in the dACC.

To our knowledge, there are no existing reports of the structural neural correlates of alexithymia in PTSD. In this study, we investigated the relationship between dACC thickness and alexithymia among PTSD patients with a history of childhood physical abuse, childhood sexual abuse, and/or childhood exposure to domestic violence. Cortical thickness was chosen as the metric of neural structure for this study because it has been shown to be a sensitive index of both brain maturational changes and the effects of early adversity [40,41]. We hypothesized that dACC thickness would be associated with alexithymia severity in PTSD patients with a history of child maltreatment.

## Methods

### Participants

Twenty-nine individuals with DSM-IV PTSD and 32 healthy controls with no history of trauma were enrolled in the present study. Thirteen PTSD patients were excluded due to: claustrophobia in the scanner (*n* = 2), PTSD symptom remission (*n* = 2), neurological disease detected post-scan (*n* = 1), left-handedness (*n* = 3), and no history of childhood maltreatment (*n* = 5). Eight healthy control participants were excluded from the analyses because of: unusable scan data (*n* = 1), history of psychiatric illness (*n* = 1), left-handedness (*n* = 3), and history of childhood maltreatment (*n* = 1). Thus, the final sample included: 16 right-handed individuals with DSM-IV PTSD and a history of childhood sexual abuse, physical abuse, or exposure to domestic violence (10 females; *M* = 34.60 *SD* = 11.51 years of age); and 24 right-handed healthy controls (14 female; *M* = 37.15, *SD* = 12.71 years of age). Participants were recruited via advertisements in the local Boston community. All subjects provided written informed consent after a full explanation of study procedures, and received financial compensation for their participation. The study was approved by the McLean Hospital Institutional Review Board, and all research activities were conducted according to the principles expressed in the Declaration of Helsinki.

Healthy adult subjects and PTSD patients were excluded from participation if they had (1) medical conditions that might affect brain structure (e.g. head trauma, loss of consciousness, seizure disorder); (2) current substance use disorder; (3) current nicotine use; (4) use of benzodiazepine or other anxiolytic, anticonvulsant, mood stabilizing, or neuroleptic medication within 4 weeks of the study; (5) history of substance abuse within the past 5 years; (6) lifetime history of substance dependence; (7) structural abnormalities on MRI scan or contraindications for MR scanning (e.g., metal implants); (8) urine toxicology positive for recent psychoactive drug use or positive pregnancy status on the day of scan. Four PTSD subjects were taking a selective serotonin reuptake inhibitor (SSRI; stable dose for more than 8 weeks), and all other subjects were free of psychotropic medication. In addition, healthy subjects had no history of DSM-IV Axis I diagnoses.

### Clinical Interviews and Measures

PTSD diagnoses were made according to DSM-IV-TR criteria as determined by the SCID-I/P. PTSD symptom scores were established using the Clinician-Administered PTSD Scale (CAPS; [42]). A doctoral-level, licensed psychologist (IMR) administered both interviews. PTSD participants experienced childhood physical abuse (12), childhood sexual abuse (10), and were also survivors of accidents (2), physical assault (3), combat exposure (1), and rape (9); twelve of the PTSD patients experienced more than one traumatic event that met Criterion A for PTSD. The mean age of onset of PTSD symptoms was 19.73 years (*SD* = 13.34).

All participants completed the Toronto Alexithymia Scale (TAS–20; [43]) and the Traumatic Life Events Questionnaire (TLEQ; [44]). The TAS–20 is the most commonly used self-report measure of alexithymia and is considered the most psychometrically valid [45]. It is a 20-item scale that consists of three subscales: Difficulty Identifying Feelings (DIF; e.g., “I am often confused about what emotion I am feeling”), Difficulty Defining Feelings (DDF; e.g., “It is difficult for me to find the right words for my feelings”), and Externally-Oriented Thinking (EOT; e.g., “Looking for hidden meanings in movies or plays distracts from their enjoyment”, which captures a tendency to focus on the external environment as opposed to introspection). Each statement is rated on a 5-point Likert scale with higher scores indicating greater alexithymia. The TLEQ is a 23-item self-report measure of potentially traumatic events including natural disasters, exposure to warfare, robbery, assault, and childhood physical and sexual abuse. For each event, respondents are asked to provide a number of times it occurred (ranging from “never” to “more than 5 times”). For this study, we classified those who reported experiencing physical or sexual abuse or unwanted sexual contact, or witnessing domestic violence before age 18, as having experienced childhood maltreatment.

### Structural MRI Data acquisition

MRI scans were conducted with a 3.0 Tesla (SIEMENS Tim Trio) magnetic resonance system using a 12-channel headcoil. A T1-weighted 3D MPRAGE sequence (TR/TE/flip angle = 2.53 s/3.39 ms/7°) was used to obtain 128 sagittal slices (256 x 256 matrix) with a slice thickness 1.33 mm and a voxel size of 1.3 mm x 1 mm x 1.3 mm.

**Fig 1:**
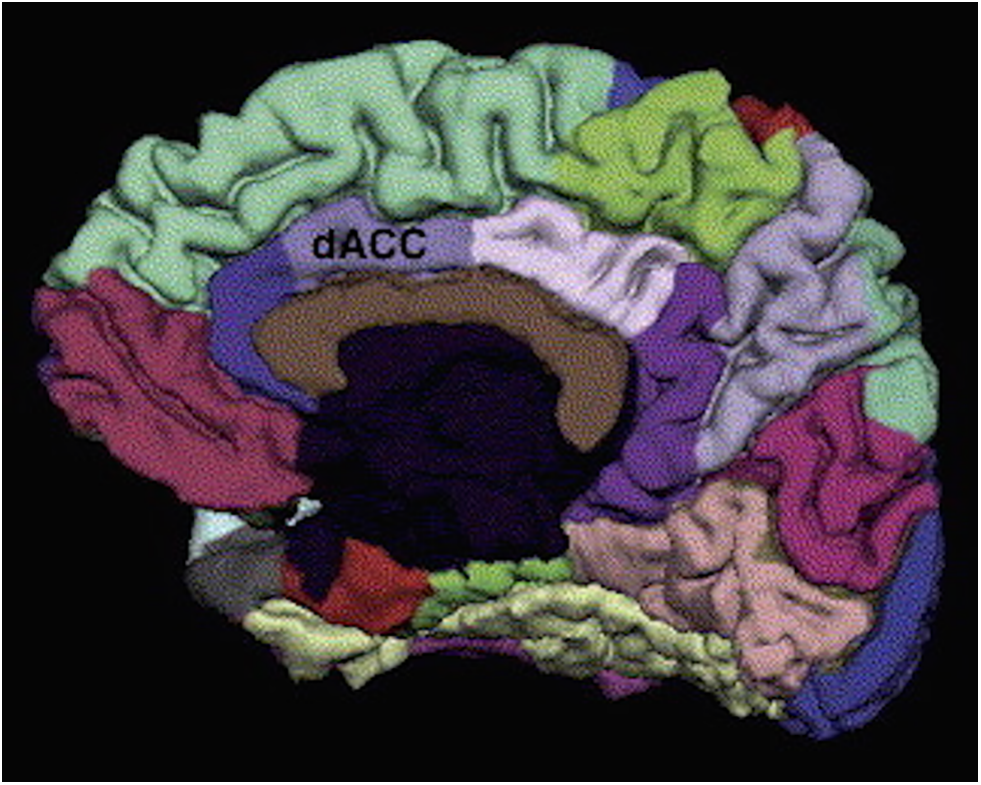
Dorsal anterior cingulate cortex (dACC) region of interest, obtained from the Desikan-Killiani FreeSurfer atlas.

Each MRI dataset underwent cortical surface reconstruction via the FreeSurfer automated cortical measurement technique [46]. Average cortical thickness measurements were taken at every point on smoothed and aligned images by averaging the distance between the pial surface and the gray-white interface. Distinct region of interest measures were calculated using the well-established FreeSurfer atlas developed by Desikan and colleagues (2006) [47]. For this study, we restricted our focus to the FreeSurfer region named “caudal anterior division of the cingulate cortex” [47] (Fig 1), herein referred to as the dACC. All participants’ FreeSurfer data were manually inspected and edited by the second author (EAO) who previously had attended an official training workshop given by the FreeSurfer developers at Massachusetts General Hospital.

### Statistical analyses

Descriptive statistics, *t* tests, and partial Pearson correlations were calculated using IBM SPSS Statistics 20.0 for Macintosh. Subjects’ TAS–20 scores and average regional dACC thickness values were compared between groups using independent group *t* tests. When Levene’s test indicated non-equality of variances across groups, *t* test degrees of freedom were adjusted using the Welch-Satterthwaite equation. Correlations with alexithymia and regional thickness focused on the à priori area in the ACC, the dACC, after partialing out the effects of sex and age. These partial correlations were run separately for each group, and are reported along with their 95% confidence intervals (CI) and power estimates.

## Results

### Group Differences

Individuals with PTSD exhibited significantly higher TAS–20 (alexithymia) scores (*M* = 52.75, *SD* = 16.46) in comparison with HC participants (*M* = 37.04, *SD* = 8.38; *t*(20.24) = 3.53, *p* = .002). Differences were driven by the affective subscales, Difficulty Identifying Feelings (*t*(17.50) = 3.67, *p* = .002) and Difficulty Describing Feelings (*t*(21.65) = 3.27, *p* = .004), but not the cognitive subscale, Externally-Oriented Thinking (*t*(38) = 1.94, *p* = .06). There were no group differences in dACC thickness (*t*(38) = 0.57, *p* = .58; Table 1).

**Table 1:** Descriptive statistics and between group comparisons (Mean ± SD or N(%)).

	PTSD Patients	Healthy Control
	N = 16	N = 24
Age, years	34.60 ± 11.51	36.15 ± 12.71
Sex, Female	10 (63%)	14 (58%)
TLEQ^a^, types of trauma*	9.44 ± 3.22	1.79 ± 1.56
TLEQ^a^, types of childhood trauma*	2.50 ± 1.10	0.00 ± 0.00
TAS–20^b^, total score*	52.75 ± 16.46	37.04 ± 8.38
TAS–20^b^, DIF^c^ subscale*	16.50 ± 7.78	9.08 ± 2.73
TAS–20^b^, DDF^d^ subscale *	15.63 ± 6.47	9.79 ± 3.72
TAS–20^b^, EOT^e^ subscale	20.63 ± 4.30	18.17 ± 3.67
dACC thickness	2.67 ± 0.20	2.64 ± 0.14
dACC^f^ thickness—right	2.67 ± 0.27	2.60 ± 0.14
dACC^f^ thickness–left	2.64 ± 0.20	2.68 ± 0.19

*p < .01; PTSD = posttraumatic stress disorder

^a^ *TLEQ = Traumatic Life Events Questionnaire*

^b^ *TAS–20 = Toronto Alexithymia Scale 20*

^c^ *DIF = Difficulty Identifying Feelings*

^d^ *DDF = Difficulty Defining Emotions*

^e^ *EOT = Externally Oriented Thinking*

^f^ *dACC = dorsal anterior cingulate cortex*

### Relationship between alexithymia and dACC thickness

**Fig 2:**
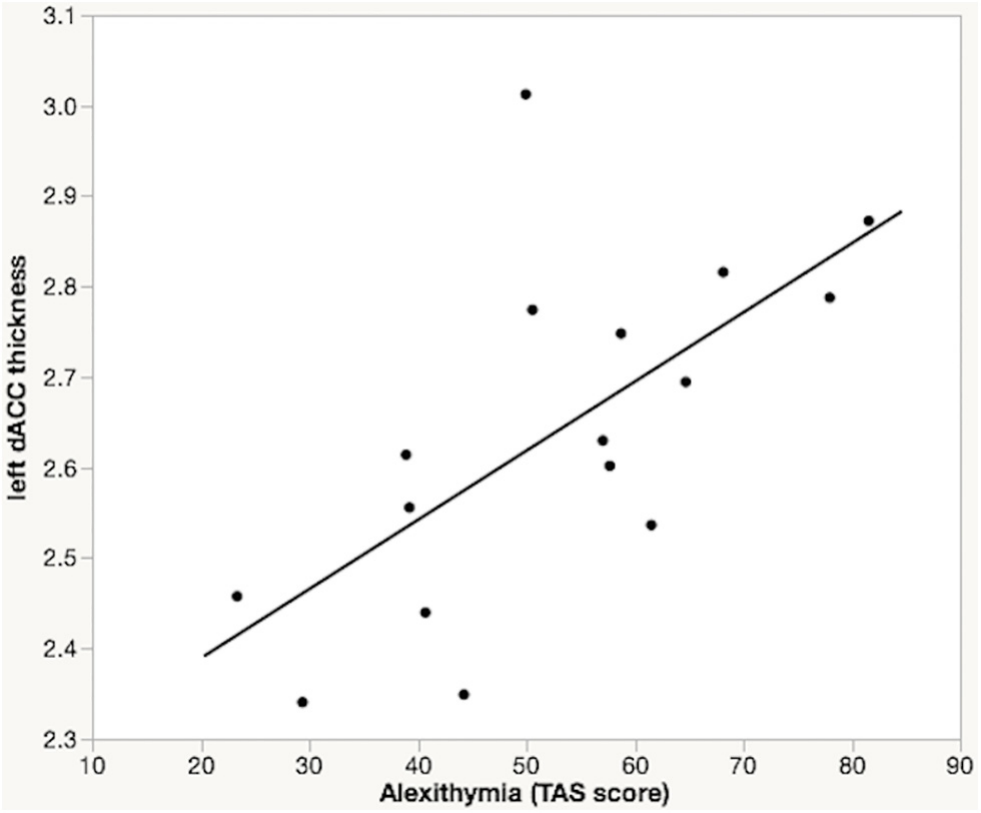
Dorsal ACC thickness and alexithymia in PTSD. Partial regression plot showing a significant association between left dorsal anterior cingulate (dACC) thickness and Toronto Alexithymia Scale (TAS–20) total score in PTSD patients with childhood maltreatment, after correcting for sex and age.

When controlling for sex and age, the correlation between TAS–20 scores and average dACC thickness was not statistically significant in the HC group (*r* = -0.19, 95% CI [-0.55–0.23], *p* = .37, power = 23%). However, the correlation of TAS–20 scores and average dACC thickness was statistically significant in the PTSD group (*r* = 0.56, 95% CI [0.09–0.83], *p* = .02, power = 76%), and this correlation differed significantly from that of the HC group (*F*(1,36) = 5.06, *p* = .03). Specifically, greater left (*r* = 0.65, 95% CI [0.23–0.87], *p* < .01, power = 89%), but not right (*r* = 0.32, 95% CI [-0.21–0.71], *p* = .22, power = 34%), dACC thickness was associated with higher alexithymia scores in PTSD patients after partialing out sex and age (Fig 2).

Follow-up analyses revealed a significant correlation between left dACC thickness and the DDF subscale (*r* = 0.72, 95% CI [0.36–0.90], *p* = .002, power = 96%), and a trend level correlation between left dACC thickness and the DIF subscale (*r* = 0.48, 95% CI [-0.03–0.79], *p* = .06, power = 62%) in the PTSD patients, after partialing out age and sex.

## Discussion

As predicted, we found greater alexithymia in PTSD patients than in healthy adult participants. Additionally, increased left dACC thickness was associated with significantly higher levels of alexithymia in PTSD subjects with histories of childhood maltreatment when controlling for age and sex. This association was particularly robust for two components of alexithymia, namely difficulty identifying and describing feelings. In contrast, individual differences in alexithymia were not significantly associated with dACC thickness in non-traumatized, healthy controls. There also was no significant difference in dACC thickness between PTSD patients and healthy adults. Overall, our pattern of findings suggests that increasing dACC gray matter thickness is a neural correlate of greater alexithymia in the context of PTSD with childhood maltreatment.

To our knowledge, the current study is the first to examine the relationship between dACC morphology and alexithymia in PTSD. A thicker dACC, which suggests a greater number and/or density of neurons, was associated with significantly greater alexithymia in PTSD patients. In addition, examination of alexithymia subscales revealed that a thicker dACC was associated with significantly more difficulty recognizing and labeling feelings. The direction of these relationships indicates that in PTSD patients, less dACC gray matter thickness is associated with better emotion awareness (evidenced as lower alexithymia). Although this may seem counterintuitive, there are many instances where less neural reserve has been associated with better emotional and behavioral functioning [48–50]. One mechanism that may underlie these relationships is experience-dependent or experience-expectant elaboration and pruning of synaptic pathways. This is a type of brain plasticity where synapses that are effectively stimulated by the environment or recruited by a psychological function are selectively maintained; in contrast, synapses that are not properly engaged or utilized are eliminated or “pruned” [51]. This refinement of synaptic pathways yields more efficient information processing and better performance across a number of cognitive and emotional domains. Thus, it is possible that PTSD patients with higher alexithymia, and hence lower ability to engage in emotional processing, are less able to recruit the dACC in a manner that permits this experience-dependent fine-tuning of cortical pathways.

Because of the cross-sectional and observational nature of the current findings, we are not able to specify the causal trajectories underlying the relationships between childhood maltreatment, PTSD symptoms, alexithymia, and increased dACC thickness. One possibility is that alexithymia may develop in response to childhood maltreatment, and altered dACC cortical thickness could result from alexithymia. This could happen if, for instance, the dACC typically undergoes experience-dependent synaptic pruning [51] in response to engaging in increasingly complex emotional identification and labeling tasks over the course of development. Failure to adequately engage in these emotional processing tasks could result in inadequate pruning of the dACC, leading to an association of alexithymia with increased dACC cortical thickness. An alternative hypothesis is that increased dACC cortical thickness could be the direct result of childhood maltreatment, and dACC abnormalities might result in alexithymia. There is evidence that early life trauma can affect postnatal brain developmental processes including synaptogenesis and pruning [52]. ACC abnormalities including increased synaptic density have been documented in rodents deprived of maternal contact [14]. Similarly, abnormal dendritic tree elaboration or synaptogenesis may underlie the association between increased dACC thickness and alexithymia in PTSD patients with a history of childhood maltreatment. Another possibility is that alexithymia and/or altered dACC thickness could predate the childhood maltreatment. For instance, it is possible that these could represent preexisting risk factors for the subsequent development of PTSD in individuals who have experienced maltreatment during childhood.

In our sample of healthy adults, there was a negative correlation between alexithymia scores and dACC thickness that was not statistically significant. This finding is in line with what has been reported by some [21] but not other [19,20] investigations, and these apparent discrepancies are likely the result of needing sufficiently large samples to detect what is a small effect in healthy adults. In the largest such study thus far, a population sample of 1,685 adults, Grabe and colleagues [22] employed a voxel-based, whole-brain query of gray matter correlates of alexithymia, which yielded the strongest correlation in the dACC. They found that total alexithymia scores, as well as subscales relating to difficulty identifying and describing feelings, were associated with significant gray matter volume reduction in a large ACC cluster and most prominently in the dorsal ACC. Importantly, even in this very large sample, the association of alexithymia with dACC morphology was statistically significant only in male participants [22]. In female subjects, the statistical threshold needed to be relaxed in order to detect an association, suggesting an even smaller effect. Overall, it seems likely that our sample of healthy adults, who did not have child maltreatment and also had limited variability in alexithymia scores, was underpowered to detect a small relationship between alexithymia and dACC thickness.

In this study, we found an association between PTSD-related alexithymia and dACC thickness only in the left hemisphere. It is possible that with a larger sample, a relationship would be detected in both hemispheres. However, the left ACC has also been previously identified as having fewer anatomical connections in maltreated individuals compared to non-maltreated individuals [34]. Moreover, there is evidence from a number of animal and human studies that early life stress can affect lateralization of brain structure and function, and that the left hemisphere is particular vulnerable to early developmental insults [52]. Overall, our findings in the context of this broader literature suggest that the functional correlates of left dACC anatomy are moderated by history of traumatic stress or early life adversity in particular.

There are several limitations and qualifications that can be made with regards to our findings. First, our sample size was too small to examine possible influences of sex or other moderating factors. As previously mentioned, prior research indicates that men and women may differ with regards to neural correlates of alexithymia [22]; similarly, there is evidence of sex differences in neural and functional correlates of early life trauma [52,53]. In light of divergent findings across research studies thus far, a larger sample is also necessary to clarify the relationship between alexithymia and ACC morphometry in healthy, nontraumatized participants. Second, as is the case for almost all studies of childhood trauma and PTSD, this study relied on retrospective data and was cross-sectional in design, so it is not possible to resolve the causal direction of our findings. Data on dACC morphometry, emotional processing capacities, and emotional functioning, before and following early life stress are needed to generate a complete picture of the relationships between these variables. Prospective, longitudinal studies of community samples are warranted to clarify the relationships between early trauma, dACC thickness, and the development of alexithymia. Overall, the nature of trauma-related changes in structural integrity of the dACC (connectedness, cortical thickness) likely relates to a complex interplay with other factors including genetic risk, sex differences, and posttraumatic stress symptoms. Finally, we should note the dACC was selected for examination in this study based on robust evidence of its involvement in alexithymia, childhood trauma and PTSD. This does not rule out involvement of other cortical and subcortical brain areas, which could be explored in future studies with larger sample sizes.

In conclusion, we found that increased left dACC thickness was associated with significantly higher levels of alexithymia in PTSD subjects with histories of childhood maltreatment. This association was particularly robust for difficulty identifying and describing feelings. These results suggest that altered dACC structure is a neural correlate of the affective components of alexithymia in PTSD. This study cannot determine whether child maltreatment led to the appearance of alexithymia in our PTSD subjects. However, this possibility seems worthy of future study based on developmental models of alexithymia whereby experiences of childhood maltreatment are thought to perturb the development of effective emotional awareness and processing abilities. Overall, this encourages future studies of alexithymia and dACC morphology in PTSD patients with and without child maltreatment, as well as longitudinal investigations on the impact of early life adversity on dACC maturation and emotional development.
